# Management of Autoimmune Encephalitis: An Observational Monocentric Study of 38 Patients

**DOI:** 10.3389/fimmu.2018.02708

**Published:** 2018-11-22

**Authors:** Stefan Macher, Friedrich Zimprich, Desiree De Simoni, Romana Höftberger, Paulus S. Rommer

**Affiliations:** ^1^Department of Neurology, Medical University of Vienna, Vienna, Austria; ^2^Institute of Neurology, Medical University of Vienna, Vienna, Austria

**Keywords:** autoimmune encephalitides, Iglon5, NMDAR, GAD 65, GAD67, autoantiboides

## Abstract

Over the last years the clinical picture of autoimmune encephalitis has gained importance in neurology. The broad field of symptoms and syndromes poses a great challenge in diagnosis for clinicians. Early diagnosis and the initiation of the appropriate treatment is the most relevant step in the management of the patients. Over the last years advances in neuroimmunology have elucidated pathophysiological basis and improved treatment concepts. In this monocentric study we compare demographics, diagnostics, treatment options and outcomes with knowledge from literature. We present 38 patients suffering from autoimmune encephalitis. Antibodies were detected against NMDAR and LGI1 in seven patients, against GAD in 6 patients) one patient had coexisting antibodies against GABA_A_ and GABA_B_), against CASPR2, IGLON5, YO, Glycine in 3 patients, against Ma-2 in 2 patients, against CV2 and AMPAR in 1 patient; two patients were diagnosed with hashimoto encephalitis with antibodies against TPO/TG. First, we compare baseline data of patients who were consecutively diagnosed with autoimmune encephalitis from a retrospective view. Further, we discuss when to stop immunosuppressive therapy since how long treatment should be performed after clinical stabilization or an acute relapse is still a matter of debate. Our experiences are comparable with data from literature. However, in contrary to other experts in the field we stop treatment and monitor patients very closely after tumor removal and after rehabilitation from first attack.

## Introduction

An association between malignancies and neurological symptoms not directly caused by the tumor itself has been described by Brouwer in 1919 and later on by Parker in 1933 (1). Thirty years ago antibodies targeting antigens Hu, Ri, Yo (anti-Hu, anti-Ri, anti-Yo) in patients with malignancies have been detected. Neurological symptoms in patients with malignancy has introduced the concept of paraneoplastic syndromes (PNS) in Neurology. Autoimmune mechanisms are hypothesized as pathopyhsiological background in PNS, as antibodies released in response to the underlying cancer are frequently found. The peripheral as well as the central nervous systems (CNS) can be affected. Encephalitis is often reported in cases with involvement of the CNS (2). Autoimmune encephalitis has to be differentiated from the PNS. It is defined as a disorder of the gray matter of the CNS that is caused by antibodies. These antibodies are targeting intracellular or surface antigens of neuronal cells in the CNS. Some of them are released in response to an existing tumor, but not restricted to malignancies—as in the case of antibodies against aquaporin-4 and myelin oligodendrocyte glycoprotein (MOG). Studies on these antibodies have revolutionized our neuro-immunological concepts. Early and correct diagnosis is highly relevant, as treatment options are available. However, the clinical spectrum is broad and it is important to look beyond the borders of neurology and to integrate other medical disciplines in our concepts of disease management. Especially psychiatric symptoms are often associated with autoimmune mediated encephalitis. Limbic encephalitis (LE) is a frequent manifestation and is defined as inflammation of (but not restricted to) the limbic region in the brain. It has been described for the first time almost 60 years ago (3), and its association with cancer was reported in 1968 (2). Typical symptoms are subacute onset of seizures, short-term memory loss, confusion and other psychiatric symptoms (4). Over the last years, LE seems to be more common as previously assumed. It is often unrelated to an underlying malignancy (5). The incidence of encephalitis of any cause (not only autoimmune mediated) is about 2–3/100.000 (6). The leading causes are infections, but in about 20% of patients, an autoimmune genesis is suspected. In a major part of the patients no definitive cause is established (6). Prevalence of autoimmune mediated encephalitis is 13.7/100.000 per 2014. Retrospective analysis of patients below the age of 35 years admitted to a German intensive care unit (ICU) because of encephalitis of unknown origin showed that 1% of all patients suffered from N-methyl-D-aspartate-receptor**-**(NMDAR) encephalitis and a British prospective population based study revealed high numbers of patients suffering from acute demyelinating encephalomyelitis (ADEM) and or voltage-gated-potassium-channels (VGCC) or NMDAR-encephalitis. Autoimmune-mediated encephalitis is more common than previously assumed (7).

Increased awareness and testing over the last years has led to a more frequent diagnosis of autoimmune encephalitis. The diverse clinical symptoms hamper diagnosis and consequently the treatment, thereby influencing the outcome and prognosis of the patients.

The aim of our paper is to propose support in the management, diagnosis, and treatment of patients with immune mediated encephalitis based on pathophysiological concepts from the literature and the presentation of patients treated at our hospital. In our patients' cohort clinical symptoms, diagnostic approaches, pathophysiological considerations for treatment, treatment options and outcomes are presented.

## Methods

Current knowledge on the background and management of autoimmune mediated encephalitis diagnosed at our center is summarized. Subsequently, patients with encephalitis treated at our hospital are presented. Diagnostics, treatment and outcome are highlighted. Diagnostic and treatment algorithms will be compared with those in literature; differences in the management will be discussed. For this monocentric study all patients with a diagnosis of autoimmune mediated encephalitis who were treated at the department of Neurology at the Medical University of Vienna between 2015 and June 2018 are reported. Immunological assessment was performed by the clinical institute of Neurology. Serum and CSF samples were investigated with indirect immunohistochemistry for surface antibodies on post-fixed rat brains and for intracellular antibodies on fixed rat cerebellum using an avidin-biotin peroxidase technique. Samples showing specific tissue staining were further examined with a commercial immunoblot assay (Ravo Diagnostika, GmbH, Freiburg, Germany) for antibodies against classic paraneoplastic antigens (Hu, Yo, Ri, CV2, amphiphysin, Ma1/2, SOX1, and GAD65). Characterization of cell surface antibodies was established using a cell-based assay (commercial kit, Euroimmun, Lubeck; in-house; HEK293T cells expressing IgLON5, mGluR1, mGluR5, GABA(A)R, AMPAR, and glycin receptor). The treating physician proposed treatment. Outcome was assessed by specialists in neurology and categorized according the modified Rankin Scale (mrs) (8). The analysis gained by the local ethics committee (Medical University of Vienna, Vienna, Austria; 1773/2016).

## Review of literature

The presentation of literature starts with the diagnostics procedure. Based on the diagnostics steps the various antibodies and their pathophysiological background causing encephalitis are will described in detail. The review ends with the proposed treatment strategies for the respective antibodies.

### Diagnostics

#### Anamnesis

The medical history of patients and a detailed anamnesis on the evolvement of symptoms and the course of symptoms is the first step in the diagnosis of patients with immune mediated encephalitis. Medical history has to include previous or existing malignancies. The detection of antibodies, nevertheless, may precede the diagnosis of a malignancy for many years. Careful and repeated tumor screening as well as tumor surveillance have to be performed. Associated malignancies are gynecological cancers like ovarian and breast cancer (9), tumors of the lungs, i.e., small cell lung cancers (10), thymoma (11), but also testicular tumors, and Hodgkin's lymphoma (12). Some of the antibodies refer to certain malignancies and vice-versa being highly relevant in the diagnostic process. Table 1 gives an overview of detected malignancies in our cohort of patients with encephalitis. Some of the patients are referred from other medical disciplines, thus an interdisciplinary management eases the appropriate tentative diagnosis. Especially referrals from psychiatrists are quite common in patients with autoimmune encephalitis. Patients are diagnosed with atypical psychosis showing clear psychotic symptoms, but diagnostic criteria for specific syndromes are not yet fulfilled (13).

**Table 1:** Initial findings in patients with autoimmune encephalitis at the time of first hospitalization.

**Ab *n***	**Positivity serum (s) liquor (csf)**	**Age in years (mean, range)**	**Sex (f in %)**	**Initial symptoms**	**MRI**	**EEG**	**CSF findings**	**Coexisting malignancy**	**Treatment**	**Symptoms to treatment (rounded; mean, range)**	**Hospitalization to 2nd line treatment (rounded; mean, range)**	**AED used**
CASPR-2 *N* = 3	S = 3 Csf = 2	64 (56-68)	0	Psychiatric, mnestic, cognitive dysfunction, speech arrest	T2 abnormalities hippocampal (67%)	Regional slowing, generalized sharp waves (33%),	total protein elevation (33%)	Neuroendocrine tumor (33%)	Pulsed steroids, IVIG, PLEX, RTX, steroid maintenance	4 months	36 days (only one patient received 2nd line therapy)	LEV, LCM, PHT, VPA (66%)
LGI 1 *N* = 7	S = 6 Csf = 4	65 (47-77)	29	Psychiatric, cognitive, mnestic deterioration, vertigo, FBDS, pilomotor seizures, muscle cramps	T2 signal alterations mesiotemporal uni- or bilateral (80%)	Epileptiform activity (43%)	Mild pleocytosis (20%), protein elevation (80%)	None	Pulsed steroids, IVIG, PLEX, RTX, AZA, steroid maintenance	5 months (4 weeks-11 months)	6 months (3 weeks-24 months)	LEV, LTG, LCM, CBZ (86%)
NMDAR *N* = 7	S = 7 Csf = 7	28 (19-41)	71	Psychiatric, cognitive, mnestic dysfunction, myoclonus, seizures, focal dystonia, catatonia	T2 abnormalities hippocampal (29%) T2 abnormalities juxtacortical (14%)	Epileptiform activity (14%) Generalized slowing (14%)	Pleocytosis (86%) Protein elevation (71%) intrathecal IgG-synthesis (43%) OCB pos (71%)	Ovarian teratoma (43%) diffuse large B-cell lymphoma (14%)	Pulsed steroids, IVIG, PLEX, RTX, CYC, MTX, bortezomib, steroid maintenance	15 days (4 days−5 weeks)	4 weeks (3 weeks-6 weeks)^*^	LEV, LCM, VPA, PHT, FBM (71%)
AMPAR *N* = 1	S = 1	20	0	Cognitive dysfunction	Unilateral T2 signal alteration hippocampal	Normal	Mild protein elevation	None	Pulsed steroids, PLEX, RTX	7 days	3 weeks	None
IGLON5 *n* = 3	S+csf = 3	71 (64-76)	100	Cognitive dysfunction epileptic seizure bilateral vocal cord palsy vertigo ataxia	T2 abnormalities: hippocampal (33%), globus pallidus bilateral (33%)	Epileptiform activity (33%)	Protein elevation (100%)	None	Pulsed steroids, IVIG, immunoadsorption RTX, AZA, CYC, steroid maintenance	69 months(2 months−10 years)	26 months (10 weeks- 28 months), one patient did not receive second line therapy	LEV (66%)
Glycin *n* = 3	S = 3	52 (48-55)	66	Paraspasticity multiple cranial nerve palsies	Hemosiderin deposits in brainstem, rostral cervical myelon, cerebellum after cerebral hemorrhage (33%)	Unremarkable (only available from 2 patients)	Protein elevation (33%), no CSF available for one patient	None	IVIG, PLEX, RTX, steroid maintenance	32 months (2 weeks-60 months)	41 days (a single patient received second line therapy)	LTG (33%)
GAD (GAD-65 *n* = 4; GAD-67 *n* = 1; GAD, GABA_A_ and _B_ = 1)	S = 2 Csf = 3	50 (27-56)	83	Vertigo, stiffness, ataxia, dysarthria, epileptic seizures, catatonia	Multiple T2 lesions cortical, juxtacortical, infra- and supratentorial (17%) Atrophy and sclerosis of the hippocampal region (17%)	Only available for one patient (17%) with normal findings	Pleocytosis (20%) OCB pos (80%) Protein elevation (40%) Intrathecal IgG-synthesis (20%) n.a. (20%)	None	pulsed steroids, IVIG, RTX, PLEX MTX, CYC, AZA, mitoxantron, intrathecally TCA, dimethyl-fumarate	12 months (2weeks - 36 months); data only available from 4 patients	47 months (9 months; 7 years)^**^	GBN, PGN (33%)
Yo *N* = 3	S = 3	58 (52-68)	100	Vertigo, dysarthria, ataxia, mucle crampi	Normal findings (100%)	n.a.	Pleocytosis (67%) OCB pos (100%) Intrathecal IgG-synthesis (67%)	Ovarian cancer (67%) Breast cancer (33%)	pulsed steroids, IVIG, PLEX, RTX, CHT/Rx	14 months (12 months, 15 months), data not available from 1 patient^***^	15 months (13 months, 17 months)	None
Ma-2 *N* = 2	S+csf = 2	66 (60-71)	100	Psychiatric, DBN, ataxia, rigor	T2 signal alterations in hippocampal region, frontobasal and basal ganglia (50%), global atrophy, small vessel disease (50%)	Normal (50%)	Pleocytosis (50%) Protein elevation(100%) Intrathecal IgG-synthesis (50%) OCB pos (50%)	Lung cancer (50%), cervical carcinoma (50%)	pulsed steroids, IVIG, CYC, steroid maintenance, CHT/Rx	12 months (2weeks, 24 months)	6 months (only one patient received 2nd line therapy)	None
CV-2 *N* = 1	S+csf = 1	51	0	Vertigo, ataxia, paraparesis	Unremarkable	n.a.	Pleocytosis, protein elevation, OCB pos.	Lung cancer	Steroid maintenance, CHT/Rx	7 months	None	None
TPO/TG *N* = 2	S = 2	31 (21-40)	50	Epileptic seizures cognitive dysfunction, psychiatric symptoms (paranoia, psychosis)	Unremarkable (100%)	Epileptiform activity (50%), generalized slowing (50%)	Pleocytosis (50%), protein elevation (50%)	None	Pulsed steroids, IVIG, AZA, steroid maintencance	42 months (24 months, 60 months)	30 months (only one patient received 2nd line therapy)	LEV, CBZ, LTG, LCM, TPM, PER (100%)

*Azathioprine (AZA), Cyclophophamide (CYC), Intravenous immunoglobulins (IVIG), Plasma-exchange (PLEX), Rituximab (RTX), Methotrexate (MTX), chemotherapy and/or radiotherapy (CHT/Rx), Levetiracetam (LEV), Carbamazepine (CBZ), Lamotrigine (LTG), Lacosamide (LCM), Topriamate (TPM), Perampanel (PER), Felbamate (FBM), Gabapentine (GBN), Pregabaline (PGN), Valproate (VPN)*.

* *The patient suffering from DLBCL had chronic immunosuppression with MMF and received RTX 9 months after initial hospitalization due to NMDAR ab positivity*.

** *3/6 patients received 2nd line therapy, medical record is only available from 2 patients*.

*** *Symptoms to treatment in PNS concerns either first or second line therapy as used for autoimmune encephalitis but not start of chemotherapy*.

#### Clinical presentations

Patients suffering from encephalitis may present with manifold symptoms including ataxia, cerebellar syndromes, movement disorders and chorea, bulbar dysfunctions, stiff person syndrome (SPS) and progressive encephalomyelitis with rigidity and myoclonus (PERM), opsoclonus-myoclonus-ataxia, seizures, down beat nystagmus, autoimmune-related retinopathy and optic neuropathy, autonomic dysfunction, neuropathic pain, peripheral nerve hyperexcitability, (atypical) psychosis and confusion, cognitive decline, sleep disorders, insomnia, and weight loss. In patients with prior history of malignancy, a new onset of neurological symptoms is suspicious for paraneoplastic syndromes. In patients with no prior history of malignancy the diagnostic procedure is more challenging and has to take into account possible malignancies (14).

#### Magnetic resonance imaging (MRI)

Brain MRI has to be performed in all patients that raise suspicion of encephalitis. In a majority of patients with NMDAR encephalitis brain MRI does not show any abnormalities at onset of symptoms (15). When abnormalities are detected they are non-specific (16). In contrast, MRI abnormalities can usually be found in patients with LE and antibody against Leucine-rich glioma Inactivated 1 (LGI1) and α-amino-3-hydroxy-5-methyl-4- isoxazolepropionic acid receptor (AMPAR) (17). Imaging studies in anti-LGI1 patients frequently show abnormalities in the hippocampal region and the temporal lobe. Bilateral hippocampal volume reduction has been reported with exception of the cornu ammonis (CA 1) region (18). Hippocampus atrophy and mesial temporal sclerosis is often observed in patients with VGCC complex antibodies, brain atrophy may be reversible in anti-NMDAR encephalitis (19–22). Infectious encephalitis (especially herpes simplex virus, HSV) is an important differential diagnosis. In most cases, abnormalities in the hippocampal region do not show contrast enhancement, diffusion restriction, or necrosis in autoimmune encephalitis, which may be helpful to differentiate from infectious encephalitis. Absence of basal ganglia involvement in temporo-mesial lesions may be suggestive of HSV (23). Patients with anti-Contactin-associated protein-like 2 (CASPR2) antibody associated encephalitis show these abnormalities to a much lesser extent (6). Nevertheless, contrast enhancement has been reported in paraneoplastic encephalitis (24). In patients with Glycin-R antibodies, two out of three patients do not show abnormalities on brain and spinal cord MRI. Abnormal cMRI results included unspecific alterations like small vessel disease (SVD) and white matter lesions (WML) (25). Brain MRI is usually normal at onset of symptoms in patients with onconeural antibodies (anti-Yo). Cerebellar atrophy might be visible after paraneoplastic cerebellar degeneration (PCD) is established (1). In half of the patients with anti-Hu antibodies abnormalities on MRI can be found (26, 27). Further patients with epileptic seizures may show temporal diffusion restriction (low ADC value) which may not necessarily indicate limbic encephalitis (28). MRI is essential for ruling out other causes; however, detected abnormalities in brain MRI might not be specific for the various antibodies.

#### Cerebrospinal fluid (CSF) and electroencephalogram (EEG)

As MRI in patients with HSV-encephalities often shows abnormalities in the temporal pole that are similar to those in patients with LE, it might be difficult to differentiate between both causalities. Thus, patients' CSF has to be analyzed. Whereas, the CSF in patients with infectious encephalitis shows pleocytosis with a moderately to highly elevated cell count and the infectious agent can frequently be detected by PCR, CSF findings are not specific for the various syndromes (paraneoplastic or not) and for respective antibodies. CSF findings can be normal, but also mild to moderate elevated cell count is seen in patients with autoimmune encephalitis (see Table 1). Over course of time, the cell count may normalize and intrathecal synthesis and oligoclonal bands (OCBs) may be present. Titres in the CSF for the various antibodies might differ or might—as seen for some cases of anti-NMDAR encephalitis—only be detectable in CSF and be more predictable for disease activity (13, 16, 29). In conclusion the CSF is helpful in differentiating between infectious and non-infectious disease (6), but can be normal and there are no distinct patterns for the various autoantibodies associated syndromes.

Similarly, the EEG may be helpful, although non-specific abnormalities are seen in infectious and immune-mediated encephalitis (6). Some EEG findings—the so called extreme delta brushes—have been reported in adults with anti-NMDAR encephalitis (30, 31).

**Figure 1:**
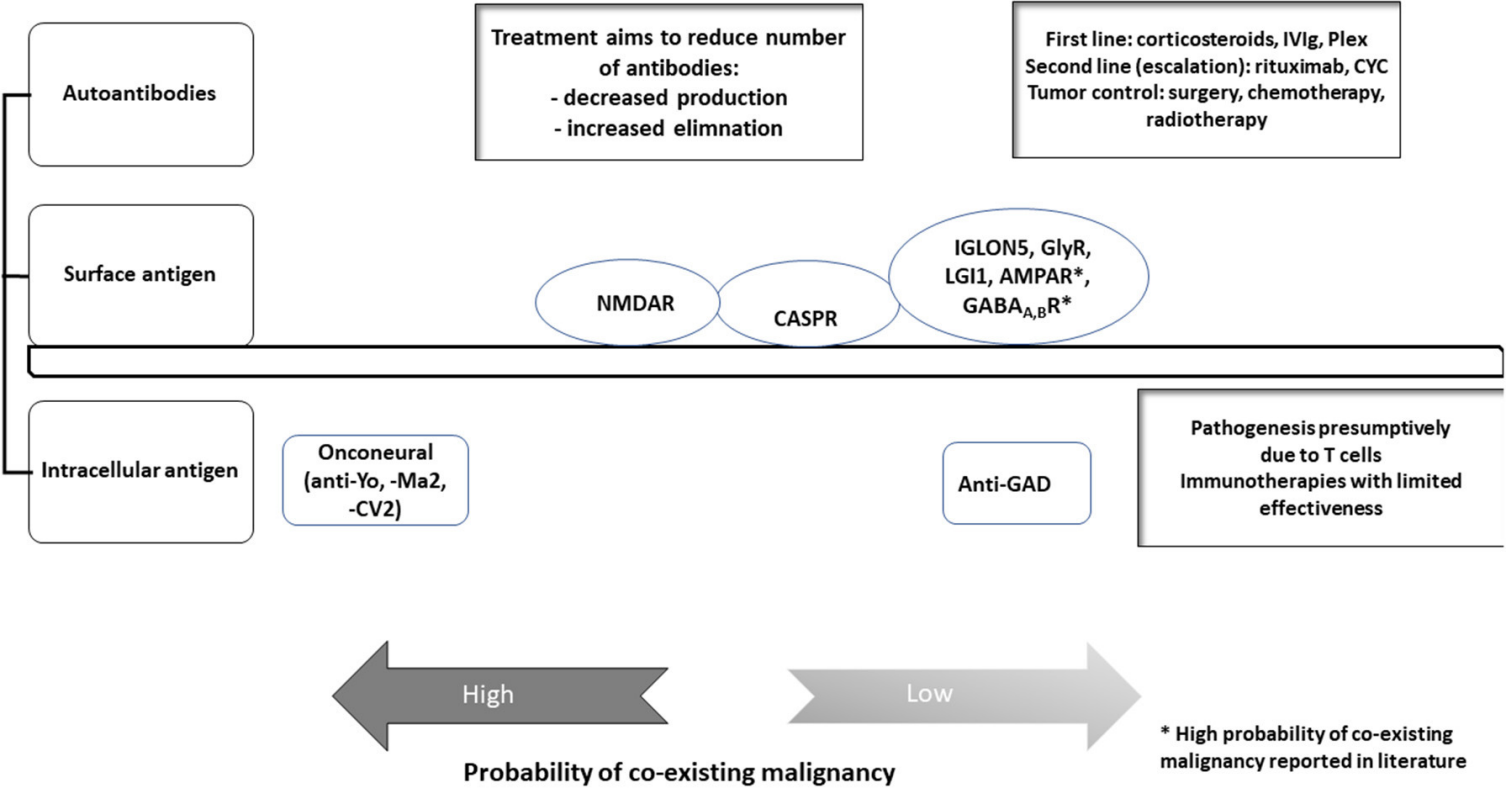
Autoantibodies in autoimmune encephalitis. This figure gives an overview on the different autoantibodies and their antigens detected in our cohort. Treatment options and probability of co-existing malignancy differs for the various antibodies in our cohort. Modified to Prüß (32).

The appropriate tentative diagnosis should take into account the results from lumbar puncture (and the correct interpretation of it) as well as the medical history, anamnesis, EEG findings, and the results from MRI. Based on the findings the suspected syndrome should be confirmed by testing for autoimmune encephalitis associated autoantibodies. Antibodies are targeting either intracellular antigens or surface antigen (neuropil antibodies). See Figure 1. Onconeural and anti-glutamic acid decarboxylase (GAD) antibodies have intracellular targets, whereas neuropil antibodies targeting surface antigens like channels e.g., VGKC—LGl1, CASPR2—or receptors e.g., NMDAR, AMPAR, GABAR, mGLuR.

### Antibodies

#### Onconeural antibodies (CV2, Ma2 and Hu, Ri, Yo)

Neuronal nuclear antibodies targeting Hu, Ri, Yo have been established for decades. They are associated with various symptoms and are various cancers. Anti-Hu antibodies (also called ANNA1—anti-neuronal nuclear antibody) were first described in 1985 (33). They are targeting intracellular antigens and are released in reply to an underlying cancer. Hu antigens (ag) can be found in malignant cells but also in neuronal cells. Currently the pathogenetic role of anti-Hu antibodies is not proven at certainty. Anti-Hu antibodies lead to a strong autoimmune response with involvement of autoreactive T cells (34).

Anti-Yo syndromes are responsible for half of all patients with PCD. Still their prevalence is low. PCD will evolve over time and may precede the detection of malignancy. Symptoms including ataxia and cerebellar dysfunction usually develop over weeks to months (1). Extracerebellar symptoms, i.e., LE, is rarer in anti-Yo patients than in anti-Hu mediated disorders. Anti-Yo antibodies target the cytoplasm of cerebellar Purkinje cells, but also other nerval structures and brain regions such as retina, dorsal root ganglia. They have the ability to fix complement and are typically IgG antibodies (35). Besides IgG also IgM and IgA antibodies have been reported. Inflamed Purkinje layer shows infiltrates of CD8+ lymphocytes, B-and T-cells and plasma cells as well as microglia activation (1). When disease progresses a massive atrophy of the cerebellum may evolve. At this stage, inflammatory cells are often missing in the Purkinje layer (36). The pathophysiological role of the antibodies is not elucidated at certainty (35). Consequently, they are not suited as marker for disease activity. Interplay of B-cells, cytotoxic T-cells and a mooted dysregulation of calcium homeostasis are pathophysiologically important.

The low number of patients limits treatment experience. Corticosteroids, plasmapheresis (PLEX) and immunoglobulin (IViG) have not resulted in convincing results. Experiences on treatments including cyclophosphamide and rituximab are anecdotal. One trial utilized tacrolimus to target cytotoxic T-cells. Whereas, the number of cytotoxic T-cells decreased dramatically, the effect was reversible when treatment was terminated. Additionally, no effects on neurological symptoms were observed (1).

Anti-Ri antibodies (also called ANNA-2) are detected primarily in patients with breast cancers and are directed against neuronal nuclear proteins. Typical manifestations are opsoclonus-myoclonus-ataxia (37).

As for other onconeural antibodies like anti-Hu, -Ri, -Yo, and CV2 does not seem to be responsible for neuronal destruction. Anti CV2 antibodies target collapsing response-mediator protein-5 (CRMP5) and are mostly associated with small cell lung cancer (11). Most frequently anti-CV2/ CRMP5 antibodies cause subacute cerebellar degeneration, followed by encephalomyelitis, limbic encephalitis, optic neuritis and retinopathy in about one in one hundred patients (38). Besides pharmacological treatment, the removal of the tumor has also only little impact on prognosis (39).

However, the survival and neurological symptoms with onconeural antibodies are associated with type of tumor and specific antibody. Although anti-Hu and CV2 antibodies lead to similar symptoms, disease outcome favors CV2 (40).

In anti-Ma2 associated encephalitis patients may present with symptoms suggestive for narcolepsy. Cataplexia and excessive daytime sleepiness result from diencephalic involvement and deficient hypocretin transmission. However, in patients with idiopathic narcolepsy anti-Ma2 antibodies have not been found (41, 42). In addition, patients presented with head drop and upper limb involvement were finally diagnosed as encephalitis with progressive muscular atrophy or as myeloradiculopathy associated with anti-Ma2 antibodies (43, 44). Brainstem, limbic and/or diencephalic involvement will lead to respective symptoms with ocular motoric disturbances, LE or symptoms suggestive for narcolepsy. CSF studies show increased protein concentration or pleocytosis, in some cases OCBs are positive. Lymphocytic infiltrates with predominantly T-cell infiltration are found in affected brain regions. Associated neoplasias are mainly testicular tumors or lung cancer. Clinical improvement was observed in patients that received a combination of tumor treatment (orchiectomy, chemotherapy, radiation) and immunotherapy (24). Stabilization or improvement has also been reported in another case series in patients receiving corticosteroids, IVIG and cyclophosphamide (45).

Whereas, the pathophysiological importance of onconeural antibodies is disputed and cytotoxic T cell may be responsible for the poor prognosis, other antibodies found in immune mediated encephalitis seem to be of great pathophysiological importance, especially for those targeting surface antigens.

Over the last years, reports of other antibodies causing encephalitis have increased tremendously. Three different targets of antibodies may be identified: 1. Receptors responsible for excitatory effects (NMDA-R, AMPA-R); 2. Receptor responsible for inhibitory effects (GAD, GABA-A, GABA-B, Gly-R); 3. Antibodies targeting channels and adhesion molecules (VGCC-, LGI1, Caspr2, IgLON5) (39).

#### Antibodies to receptors mediating excitatory effects

##### Antibodies against the n-methyl-d-aspartate receptor (NMDAR)

Antibodies against the receptor of NMDAR were described in 2005 in four female patients presenting with psychiatric symptoms for the first time. They responded to immunotherapy and/or ovarian teratoma resection. Incubation of patients' sera with rat hippocampal neuron cultures showed intense immunolabeling with ags localized in the molecular layer of the hippocampus (46). In 2007, the target auto-ags were identified as located in the extracellular domain of the NMDAR subtypes 1 (NR1) and 2B (NR2B), and to a lesser extent to the NR1 and NMDA-R subtype 2A(NR2A) as conformational epitope (47). The main cellular mechanisms accounting for the stereotypical course of anti-NMDAR encephalitis are: (1) Patients' CSF anti-NR1 antibodies or purified IgG reduce surface NMDAR protein and NMDAR cluster density in a titer dependent manner compared to healthy controls. (2) Additionally, patients' antibodies reversibly and specifically reduce NMDAR from excitatory synapses, and thereby not affecting the total number of excitatory synapses. (3) This process is mediated partly by capping, crosslinking and internalization of NMDAR independent from complement activation (48). Established treatment strategies comprise various forms of immunotherapy (corticosteroids, IViGs, plasmapheresis PLEX, rituximab, cyclophosphamide).

##### Antibodies against the α-amino-3-hydroxy-5-methyl-4-isoxazolepropionic acid receptor: (AMPAR)

Anti-AMPAR antibodies have first been described in 2009 49. AMPAR belongs to the glutamate (Glu) receptor, and is responsible for excitatory synaptic transmission in the brain. The antibodies target one of the subunits of the GluR: GluA1 or GluA2. GluA1 and GluA2 are surface ags. The binding to the receptors leads to an internalization of the receptors (50). Its importance for memory, learning and synaptic plasticity is well-characterized (51). Psychosis is quite often the initial symptom and clinical presentation of patients is similar to those in anti-NMDAR encephalitis. An association with breast cancer, tumors of the lungs (e.g., small cell lung cancer) and the thymus has been observed (52, 53).

#### Antibodies to receptors mediating inhibitory effects

##### Antibodies against glutamic acid decarboxylase (GAD)

GAD is the enzyme needed in catalyzing the decarboxylation of glutamate to γ-aminobutyric acid (GABA). Anti-GAD antibodies are frequently detected concurrent with other antibodies—most frequently with antibodies against GABAR (54). The two receptors on which GABA acts as an inhibitory ligand in the CNS are GABA_A_, an ionotropic receptor, and GABA_B_ a metabotropic receptor. Of the two isoforms of the enzyme, GAD 65 and 67,the first is located mainly in synaptic vesicles and synthesizes GABA in an activity dependent manner, whereas GAD 67 is located in the cytosol; is constitutively active and accounts for a steady state of basal GABA level (55, 56). GAD antibodies are associated with various neurological diseases including stiff person spectrum disorders (SPSD), cerebellar ataxia, PERM, LE, epilepsy, down beat nystagmus, autoimmune-related retinopathy and optic neuropathy (ARRON syndrome) (57–59). Anti-amphipyhsin antibodies are commonly detected together with anti-GAD antibodies. Together these are the three auto-antigens for cerebellar ataxia, SPS and Batten's disease (60).

Anti-GAD titres in neurological diseases are usually substantially higher than in patients with diabetes mellitus type 1 (DMT1), though there is an overlapping range. Whether the ability to cross the blood brain barrier (BBB) is titer dependent is speculative (61), but might be an explanaition why low titres causing DMT1 and higher titres causing CNS symptoms. Vice-versa high titres in SPS can cause damage to the neuroendocrine beta islet cells, and over the course up to 30% of SPS patients develop autoimmune diabetes mellitus (62). GAD antibodies found in neurological diseases have a different epitope specificity than in patients with DMT1 (63, 64). If GAD antibodies are directly pathogenic or whether they are just an epiphenomenon for autoimmune disorders that are mediated by CD4+ T cells is still a matter of debate (65–67). Electrophysiological studies have led to SPS- like symptoms and cerebellar ataxia in rats after injection of sera from patients with antibodies against GAD into rat cerebellum and lumbar para-spinal region (64). It has also been shown that passive intrathecal transfer of IgG from SPS patients can cause SPS like motor symptoms in the rat model (68), elucidating pathophysiological relevant antibodies in SPS patients. Despite its intracellular location the intraperitoneally passive transfer of human IgG against synaptic amphiphysin in a rat model evoked symptoms analog to human SPS supporting a direct pathogenic role of the antibodies (69). A positive therapeutic effect after IVIG therapy in patients suffering from SPS has been reported before (70). Antibodies against GAD are usually not associated with tumors. However, patients with a concurrent antibodies to GAD directed against cell surface antigen seem to have a 7-fold higher risk of having an occult neoplasm (71, 72). Paraneoplastic SPS is mostly accompanied by anti-amphipysin antibodies and associated mainly with breast cancer (9). In some patients with endocrine autoimmunitiy the presence of GAD65 antibodies might precede the onset of a neurological disorder (73).

A randomized placebo-controlled trial of patients with SPS found no significant positive effect after the administration of rituximab over a period of 6 month though 4 patients improved markedly (74).

##### Antibodies against γ-aminobutyric acid (GABA)-receptors

Anti-GABAR antibody block the inihibitory effects mediated by GABA-R. There are two different forms of receptors: GABA_A_ and GABA_B._ They are usually associated with LE (GABA_B_) or refractory seizures (GABA_B_). Whereas, GABA_B_-R antibodies are frequently associated with tumors, this association is less commonly seen in patients with antibodies against GABA_A_-R. They usually respond to immunotherapy (75).

##### Antibodies against glycine receptors (GLyR)

In 2008, antibodies against GlyR were discovered in the serum of a patient diagnosed with PERM (76). GlyRs consist of alpha 1–3 and beta subunits (GLRA1-3). The alpha 1 and beta subunits of the GlyR are expressed abundantly in the pontine region, medulla oblongata and upper spinal cord (25, 38). The role of antibodies directed against GlyR A2 and GlyR A3 as intracellular epitope is unclear (25). The binding of Gly to its receptor leads to chloride influx and hyperpolarization of the postsynaptic cell. Whether the receptor internalization and the direct inhibition of the GlyR contributes to pathology remains unclear. In patients with paired serum-CSF samples the GlyR antibody titer was more prominent in the sera (25, 77). Patients may present mainly with muscle spasm, stiffness, rigidity, and myoclonus. In addition, cranial nerve involvement, excessive startle, walking problems, and cognitive deterioration are frequently associated symptoms. There is an association with neoplasms in up to one out of four patients. After treatment of cancer, neurological symptoms improved. Interestingly a major part of patients seem to improve with immunotherapy and became independent in daily activities (25, 77). PERM, a condition already described in the 1970s, can be distinguished from SPS by its progressive course, brainstem, cranial nerve and long tract involvement (78, 79).

#### Antibodies targeting channels and adhesion molecules

##### Antibodies against the voltage gated potassium channel-complex (VGCC-complex): Contactin-associated protein-like 2 (CASPR2), Leucine-rich, glioma Inactivated 1 (LGI1)

The discovery of CASPR2 and LGI1 as main auto-ags of the VGCC complex led to a better understanding of channelopathies. Clinical manifestation and responsiveness to steroids differs between the two antibodies (39, 40). CASPR2 is a cell adhesion molecule and can be found in the hippocampus, cerebellum, and in the juxtaparanodal area of myelinated nerves in the CNS and PNS. It is a transmembrane protein with a small intracellular and a large extracellular domain and belongs to the neurexin IV (Nrx-IV) superfamily. In the juxtaparanode region, CASPR2 together with TAG1 (a neuronal cell adhesion molecule) and protein 4.1B organize and localize Kv 1.1/Kv 1.2 channels (53, 80, 81). Antibodies directed against CASPR2 are predominantly of the IgG4 subtype and importantly do not cause internalization of the protein and lack crosslinking as seen in other types of encephalitis (48, 82, 83). Antibodies to VGCC may be directly pathogenic and may disrupt the cell to cell interaction (84). The largest retrospective study of patients with CASPR2 antibodies showed that the majority of patients are males with a median age of 66 years. The most prominent symptoms are cognitive disturbance followed by seizures and peripheral nerve hyperexcitability. CSF was normal in more than two thirds of the patients and about 70% had a normal brain MRI. All patients had serum antibodies against CASPR2. Patients with a tumor might have low CSF titres or no antibodies detected in CSF by immunohistochemistry due to the primary peripheral involvement. Tumor prevalence may account to up to one fifth of patients and are in most cases thymomas or small cell lung cancers. In patients with a tumor surgery, the concomitant chemotherapy led to complete neurological remission. Relapses occurred in 25% of the patients, the earliest 8 months after the initial episode and symptoms were mostly similar than in the initial episode. Interestingly, Morvan Syndrome—characterized by peripheral nerve excitability, encephalopathy, autonomic dysfunction and sleeping disorder—was also associated with channelopathies (85). Response to treatment in patients with Morvan's Syndrome took longer than with other presenting symptoms but taken together 72% of patients became independent in daily activities at a median follow up of 36 months, whereas 21% of patients were treated with immunotherapy other than first line therapy (84, 86). Serum cut off titres of ≥1:200 showed good sensitivity and specificity for the diagnosis of CASPR 2 encephalitis especially when a brain MRI was performed in addition (87).

LGI1 stabilizes the compound between ADAM22 (a disintegrin and metalloproteinase domain) and ADAM23 close to VGCC in the presynaptically and to AMPA-R postsynaptically (88). There are about 300 reported patients with encephalitis associated with LGI1 antibodies resulting in an estimated incidence of 0.83/million (17, 86). The most common initial symptoms seem to be epileptic seizures and cognitive deterioration, though during the course of the disease more than 80% of the patients develop seizures (17, 89). Tumors are present in up to 20% of patients (17, 18, 90, 91). Further common associated symptoms are insomnia and dysautonomia. Faciobrachial dystonic seizures (FBDS) are reported in almost 50% of patients and are a characteristic (92–94). FBDS do not seem to respond to antiepileptic drugs but to immunotherapy (89, 95). Hyponatremia is found in 65% of the patients. Two out of three patients show hippocampal alterations in MRI at presentation, mostly unilateral and three out of four patients show normal CSF findings in the lumbar puncture.

First-line treatment response rate is effective in 80%, and improvement started with decrease in seizures and improvement of cognitive functions. Eighty-six perecnt had persistent amnesia for the initial disease and life events during the disease as well as retrograde amnesia representing as lack of memories for vacation. Relapses occurred in 35% (17). Imaging studies in LGI1 patients showed hippocampal volume reduction in all segments besides of the CA1 region. The duration of FBDS correlated inversely with the volume of the right pallidum (18). Rituximab seems to be safe and effective even in a later course of the disease in patients with LGI1 antibody encephalitis (96). Patients with LGI1 antibodies seems to have poor memory recovery probably because of structural damage due to hippocampal atrophy (97). Though as it has been observed in patients with FBDS the initiation of immunotherapy may prevent cognitive deterioration (95).

##### Antibodies against IglON5

Anti-IglON5 antibodies were first reported in eight patients with predominantly atypical sleep disorders in 2014 (98). IgLON5 is a neuronal cell adhesion molecule with unknown function. IgLON5 antibodies are accompanied by phospho-tau deposits in subcortical areas mainly in the hypothalamus, brainstem tegmentum and upper spinal cord (99). The hallmark of anti-IgLON5 associated encephalitis is parasomnia involving REM and non REM sleep with stridor, abnormal sleep behavior, e.g., patients mimic activities of daily living, and sleep apnoea (98, 100, 101). Four core symptoms have been reported in the largest published case series: (1) predominantly sleep disorder (2) bulbar dysfunction (3) progressive supranuclear palsy (PSP) like syndrome (4) cognitive deterioration or major neurocognitive disorder (102). The disease is strongly associated with the HLA-DRB1^*^1001 and DQB1^*^0501 alleles linking neurodegeneration with the immune system (101, 102). There is no ensured paraneoplastic origin though the presence or history of cancer which may not be causal has been described in patients (103). Different to NMDAR and AMPAR, anti-IgLON5 antibodies cause an irreversible downregulation of the surface protein. This is caused by IgG1 antibodies in a time dependent manner and may be a major reason why patients do not respond fully to immunotherapy (83). Antibodies to IgLON5 lead to irreversible internalization of the IgLON5 protein. Consequently, the long time period between symptom onset and start of immunotherapy may be responsible for the low effects of treatment. Besides IgG1 also IgG4 have been reported (103). It is still unclear if patients with predominantly IgG4 rather than IgG1 or the HLA-type is associated with better outcome.

##### Hashimoto encephalopathy (HE)/Steroid responsive encephalopathy associated with autoimmune thyroiditis (SREAT)

HE/SREAT was first described by Brain et al. (104). Under the aspect of unclear underlying pathophysiological mechanisms, there is discussion about the right terminus for the disease. As HE/SREAT is not necessarily associated with thyreoiditis, and not all patients respond to corticosteroids (105, 106). HE/SREAT is a diagnosis of exclusion and can be considered under certain conditions after alternative causes have been ruled out (14). Patients diagnosed with HE/SREAT frequently show elevated CSF protein or CSF pleocystosis. Cranial MRI is usually unremarkable. EEG may show unspecific abnormalities but does not show typical patterns like in patients with anti-NMDAR encephalitis. Generalized slowing is observed in patients, in some with lateralized slowing or intermittent rhythmic slowing in frontal or occipital regions with epileptic activity in some cases and improvement of EEG pattern under therapy while follow up (106–108). HE/SREAT is not necessarily associated with hypo- or hyperthyroidism but serum anti-thyroid antibodies seem to present ubiquitary in these patients whereas CSF thyroperoxidase (TPO) and thyroglobulin (TG) antibodies are rarely positive (108). The thyroidea stimulating hormone (TSH) levels can be normal (108). Antibodies-titres and CSF protein seem to decrease concordantly to clinical improvement (106, 109), though the levels of CSF antibodies seems to be independent of the clinical stage of the disease (105). Patients with initial coma may have relapses more often than those without coma (108). Outcome is generally favorable and response to first line corticosteroids is good. Up to 18% of the disease-free population in an U.S. collective have TG or TPO antibodies. Antibodies are detected more often in older white females and the occurrence of TG antibodies in > 50% goes together with the appearance of TPO antibodies and vice versa (110). If anti-thyroid antibodies are causal to the disease or if they are just an epiphenomenon is not elucidated so far. Nevertheless, anti-TPO monoclonal antibodies bind to astrocytes (111).

### Prognosis of autoimmune mediated encephalitis

The various antibodies and the antigens they are targeting have major influence on prognosis. Patients with antibodies against intracellular antigens (see Figure 1) have worse prognosis (112). Pathomechanisms involve quite often cytotoxic T cells that are responsible for neuronal destruction (113). In patients with antibodies against surface antigens outcome may be better, as immunotherapies might be more effective in those patients. Antibodies titres as well as the epitope are of the utmost importance in those patients. Outcome in patients with NMDAR encephalitis might be better than in those with AMPA-R. In patients with antibodies against onconeural structures malignancies will be found, as these antibodies are well-established markers for PNS. Contrary, not all patients with antibodies against surface antigens will have neoplasias. However, a screening for neoplasias and malignancies is obligatory. Whereas, in patients with onconeuronal antibodies associated disorders outcome is generally poor even after removal of the tumor, the disease course in encephalitis might be improved in dependency of the various antibodies and underlying tumors after removal of them.

### Treatment

The appropriate treatment aims to stop the immunological processes being causal for the disease and to treat sequelae of encephalitis. Existing malignancies should be removed and treated adequately as soon as possible to eliminate the causing “antigen.” Additionally, immune-suppressive treatment should be started. Treatment options include corticosteroid, IViG treatment, plasmapheresis, rituximab, cyclophosphamide. For some patients a combination is needed (114).

Immunotherapy is of vital importance, however, also treatment of sequelae such as epileptic seizures is a major concern.

### Conclusion

Some patients do not show immune mediated antibodies. In those patients the application of nuclear medicine diagnostics—especially in patients with unremarkable magnetic resonance imaging (MRI)—, and cerebrospinal fluid (CSF) analysis allows diagnosis of autoimmune encephalitis (14, 115, 116). Consequently, in patients with new onset of atypical psychosis and negative antibody-testing CSF analysis is recommended (117–121). Diagnosis of autoimmune encephalitis remains challenging not less than establishing an appropriate therapeutic concept for each patient. Hereto identification of prognostic factors as figured out in anti-NMDAR encephalitis may alter therapeutic strategy (15). Biomarkers like neurofilament light chains (NFL) and phospho-tau may offer future strategies for disease monitoring acting as a surrogate for disease activity (122). Still, there are unanswered questions regarding etiology. An infectious link was proposed in anti-NMDAR encephalitis, as patients with acute symptoms after HSV-encephalitis often show antibodies against NMDAR. In addition, an association between non-encepahlitic HSV-1 infection and NMDAR-encephalitis has been proposed based on results of a case-cohort study (123). CXCL 13 has been shown to be useful to identify acute neuroborreliosis and its utilization as biomarker for treatment response in patients with NMDA-R-encephalitis may offer future strategies (124, 125).

## Results

Thirty-eight patients diagnosed with autoimmune encephalitis were included in our analysis. Sixty-one percent were female. Mean age was 48 years (ranging from 19 to 77years), and was similar for sexes (females: 50 years [19–77] and males 53 years [21–77]). The youngest patients (mean age) were among the NMDAR, AMPAR and TPO/TG subgroup, and age was highest for patients with IGLON5 (*n* = 3, 71 years [64–76 y]), followed by Ma-2 with 66 years (*n* = 2, 60 and 71 years), LGI1 (*n* = 7, 65 years, [47–77 y]). Antibodies against NMDAR and LGI1 were detected in 7 patients and were the most common ones. Six patients had antibodies against GAD (5 patient's GAD-65, 1 patient GAD-67). One patient with anti-GAD-67 antibodies also showed antibodies against GABA_A_ and GABA_B_. Concomitant cancers were observed in 11 patients. All patients with anti-Yo, anti-Ma-2 and anti-CV2 antibodies and 57% of patients with NMDAR antibodies as well as one patient with CASPR2 antibodies had coexisting malignancies. In two patients with Ma-2 abs preceded tumor diagnosis, and in one patient with CV2 abs preceded tumor recurrence.Four patients with NMDA-R encephalitis had a malignancy (three women with teratoma, and one male with B cell lymphoma).

Patients with NMDAR encephalitis presented most frequently with neuropsychiatric symptoms (agitation, confusion and hallucinations). LE was the most common syndrome in patients with LGI1 and CASPR2 encephalitis. Mnestic and cognitive deficits but also seizures were common as initial symptoms in those patients. Spasticity and ataxia were the leading symptoms in patients with GAD-65 and GlyR antibodies. In one patient with GlyR antibodies cranial nerve involvement was reported. Patients with ant-IgLON5 syndrome do not show distinct patterns of symptoms (see Table 1). All patients with anti-Yo antibodies were diagnosed with PCD and presented with ataxia. MRI abnormalities were detected in 47.4% of all patients and differed for the various antibody associated syndromes ranging from 0% for CV-2 (*n* = 1), SREAT (*n* = 2), Yo (*n* = 3) up to 100% for Ma-2 (*n* = 2) as well as the patient with antibodies against AMPAR. Whereas in LGI (*n* = 7) abnormalities were detected in 86% (*n* = 6), the rate was 43% (*n* = 3) for NMDAR (*n* = 7). Most prominent abnormalities were seen in the hippocampal and mesiotemporal region. These radiological findings correlated with symptoms (psychosis, cognitive, and mnestic deficits).

EEG abnormalities were either general slowing or epileptiform activity and were seen in 31.4% of the patients. Anticonvulsant drugs were used in 20 patients. Out of 25 patients who received second line therapy 7 patients (NMDAR: 2; LGI1: 1; AMPAR: 1; Ma-2: 1, Yo: 2) did not receive AED. Four of those patients (NMDAR, LGI1, AMPAR) recovered well (mRS ≤ 2). Most common AED used were Levetiracetam, Lacosamide and Lamotrigine. Ninety-two percent of patients with documented seizures in the subacute phase still had AED 12 months after start of immunosuppressive therapy. AED were most likely used in the subgroups with CASPR2, LGI1, NMDAR, IGLON 5, and TPO/TG antibodies (≥66% of patients).

All patients with anti-NMDAR encephalitis and coexisting ovarian teratoma underwent surgery within 7 days after detection. Two out of these three patients had a favorable outcome (mRS 0). A patient with paraneoplastic anti-Ma2 brainstem-encephalitis was diagnosed with lung cancer several years before neurological deterioration indicated tumor recurrence. The patient received chemotherapy and radiation. About 12 months after onset of neurological symptoms antibody titres decreased. Clinical improvement was recognized though the patient is still not able to walk unassistedly. Similarly, a female patients with anti-Yo antibodies was treated for breast cancer (surgery, chemotherapy, radiation). Antibodies were detected after onset of ataxia. The other patient with Ma-2 suffering from limbic encephalitis received chemotherapy which started a few weeks after diagnosis. Immune-suppressive therapy with cyclophosophamid was started but was stopped after five cycles as the patients symptoms did not improve. A patient with CV-2 mediated brainstem encephalitis has recently been diagnosed and tumor management has been initiated. All patients with anti-Yo antibodies underwent surgery. As the time interval between surgery and start of immunosuppressive therapy is unknown we cannot report further details in this context. Outcome for patients with onconeural antibodies is worse than for those with surface antigens. None of the patients were independent in daily activities. *mrs was* ≥3 for all patients with onconeural antibodies.

Most administered treatment were IVIG (2 g/kg bodyweight over 5 days, up to three times) and pulsed steroids (1 g methylprednisolone for 5 consecutive days, followed by 75 mg oral dose tapered over 12–20 weeks) in 26 patients. Eleven patients received plasma exchange (cycles of up to 11 plasmapheresis and up to two cycles) or immunoadsorption. Twenty-four patients received ≥3 different immunotherapies and 12 patients were treated with ≥4 different immunomodulatory therapies. Only 2 patients responded well to first-line treatments (mRS score ≤ 2), and no escalation therapy was initiated. Twenty-five patients received second line therapy. Most common second lines treatments were rituximab in 22 cases (up to three times in the acute treatment, 375 mg/m^2^ body surface), cyclophosphamide (750 mg) in 7 cases, methotrexate (10 mg weekly up to three times) and azathioprine (1.5 mg/kg bodyweight) in 3 cases. Twelve patients received an escalation treatment consisting of more than one second line treatment. Most commonly, rituximab and cyclophosphamide were given as add on (see Table 1).

Patients with at least 6 months of follow up were looked at in detail. Stratified by modified ranking scale patients that score 0 points had NMDAR (66%) and AMPAR antibodies (33%). We stopped treatment after initial application of rituximab (2 times, 14 day interval), after 13 and 20 months in those patients. Both patients with NMDAR antibodies underwent ovarian teratoma resection. Seven patients scored 1 point. All patient had treatment with rituximab initially (2 times, 14 day interval), and two did not receive further immunosuppression (both LGI1 antibodies). Two patients improved under first line therapy and are currently under observation (CASPR2 and LGI1). Two patients received chronic second line immunosuppression (non-paraneoplastic NMDAR and HE) and another patient is under chronic immunosuppression for 2 years now and will soon be reevaluated (LGI1). Of 5 patients scoring 2 points three received chronic second line immunosuppression (2 IgLON5, 1 LGI1), one patient with GlyR mediated SPS stabilized received IVIG with mild stabilizing effect but without significant improvement and refused second line therapy and another patient with GAD 67 antibodies improved distinctly under IVIG which was stopped after 4 cycles and is currently under observation.

## Discussion

Reports on autoimmune mediated encephalitis have increased tremendously over the last years. This development was mirrored in our institution by a large number of newly diagnosed and treated patients at our institution. The increased awareness may have led to more testing for autoantibodies and consequently to more diagnosed patients. Testing for antibodies in serum is easily available. Still sensitivity of antibody testing may be higher in CSF than in sera as reported for NMDAR encephalitis (29) and thereby diagnostic pathway is more invasive. Moreover, in consistency with literature (29) we observed that the clinical course correlates well with the NMDAR antibodies titres in *the* CSF of patients. This might be of importance especially in comatose patients when clinical neurological assessment is limited. For patients with suspicion for LGI1, CASPR2, IgLON5, GAD, and Glycine mediated disorder testing of serum may be sufficient, but as symptoms are unspecific we test antibodies in serum as well as in CSF.

Diagnosis is hampered by the presentation of very unspecific symptoms (118, 126), but early diagnosis and treatment initiation are essential in the management of autoimmune encephalitis. We have observed wide disparity in the latency of diagnosis and treatment in our cohort. Patients diagnosed with anti-IGLON5 syndrome followed by HE and GlyR antibody associated encephalitis had the biggest latency in receiving a diagnosis and treatment with immunosuppressive agents. The highly variable interval in “first symptoms to immunosuppressive treatment” may be explained by the increasing awareness for NMDAR and LGI1 encephalities, whereas diagnosis might be challenging for HE/SREAT, or antibodies testing for IGLON5 or GAD-67 was not available until recently. One of our patients with antibodies to IGLON5 was diagnosed after 7 years of bilateral vocal cord palsy. Interestingly, he improved under immunotherapy. A case with CASPR2 encephalitis in our cohort had a very long disease course and similarly he improved under treatment. Both cases may suggest that even after a long period treatment should be initiated, and seems to be more effective than assumed (103, 127, 128).

First line therapy was initiated within the first few days after hospitalization for most of the patients (especially for those with NMDAR and LGI1 encephalitis). First-line therapies were pulsed steroids and IVIG. Treatment was escalated to PLEX in patients with antibodies against surface epitopes and who did not respond to high dose steroids or IVIG. Depending on the patient's clinical neurological condition during PLEX or after 6 cycles of PLEX second line therapy was initiated which is more or less in line with previous recommendations (16). Initiation of second line therapy has evolved earlier in the course of the disease over the last 4 years and may have improved the disease course. The first choice of second line immunotherapy was rituximab in most cases, in some cases simultaneously with cyclophosphamide. In patients with onconeural antibodies second line therapy was mostly cyclophosphamide and to a lesser extent rituximab. We figured out that the initiation of second line therapy is highly dependent on the notification of the antibodies status. None of our patients received second line therapy without diagnosis evidenced by respective antibodies. A prolonged disease course with no clear improvement was the basis for the decision to initiate escalation therapy in most of the patients. When escalation treatment should be started is a matter of discussion, as there are no clear guidelines. Maintenance therapy with rituximab has been established for varying duration. We re-evaluated immunosuppression with rituximab after 4 to 6 cycles (2–3 years after initiation). There is need to implement scales with sufficient sensitivity and other testing modalities (e.g., autonomic testing) to monitor patients in the acute phase but also in therapy surveillance.

If patients showed clinical improvement, regained autonomy in daily activities, CSF has normalized, MRI did not show new alterations and antibody status became negative than we usually discontinue chronic immunosuppression and arrange follow up controls in 6 months intervals. In cases with coexisting tumor we stop immunosuppression in our NMDAR patients 1 year after tumor removal, even if they had had second line therapy. This is not absolutely conform with the proposed management (16). Termination of immunosuppressive therapy needs to be discussed in each individual case and cannot be recommended without reservation for all patients with autoimmune encephalitis or even with the same distinct antibodies, although all of our patients had a monophasic course of the disease until now (even in those patients with second line treatment). In patients with stiffness and PERM besides immunotherapy also symptomatic treatment with high dose oral and intrathecal triamcinolone-acetonide that markedly reduced stimulus-evoked jerks, reduced rigidity and muscle spasms is of importance. Treatment response to immunotherapy in patients with cerebellar ataxia was markedly worse than in patients with SPS. On the other hand, patients with non-paraneoplastic ataxia and seropositivity for GAD65 antibodies respond better to immunotherapy than patients with coexisting malignancy. In those patients early treatment initiation is of the utmost importance (129, 130).

Despite the small number of patients almost all our patients with antibodies against LGI1 and NMDAR developed epileptic seizures which disappeared as they recovered. Seizures in our patients developed early in the course of the disease, although literature report manifestations in every stage of the disease (131). Seizures as first symptom of anti-NMDAR encephalitis are more common in men. Since we only have one male patient who did not develop epileptic seizures we cannot confirm that seizures manifest in men more frequently (132). Five patients with antibodies against LGI1 developed epileptic seizures. FBDS were observed in a single patient. Seizure control was achieved by early immunotherapy whereas cognitive deficits persisted in 80% of our patients, similarly to reports from literature (17, 133). All patients with seizures as initial symptom or in the subacute phase received AED and immunosuppressive therapy simultaneously. Most patients, especially those with LGI1 antibodies stabilized soon and did not suffer from further seizures. Patients with NMDAR encephalitis had seizures mostly as initial symptom or in the subacute phase often associated with autonomic dysregulation and need for intensive care treatment but not after clinical stabilization. Taken together we cannot link clinical improvement to AED. Some patients with limbic encephalitis did not suffer from seizures, maybe because of fast initiation of immunotherapy, did not require AED and improved markedly over the course of their disease.

Tumor screening was performed in all patients with anti-NMDAR encephalitis. In our female patients screening for teratoma was done either with computer tomography (CT), pelvic ultrasound or as recently reported by MRI (134) or a combination of these modalities. The removal of the ovaries in patients with teratoma is aimed to be conducted immediately after detection and diagnosis of NMDAR encephalitis as not only the severity of symptoms but also early initiation of immunotherapy and early teratoma resection predict good outcome (15). Tumor surveillance is of utmost importance as the relapse rate and prognosis depend on the tumor status (15), and tumor work up in yearly intervals should be performed (16). Besides teratoma, B cell lymphoma was diagnosed in one of our male patients. Detection of NMDAR antibodies preceded diagnosis in this patients. Whether there is a pathophysiological relation remains unclear. EBV is of importance in the pathophysiology of B cell lymphoma, and recently a case with anti-NMDA-R encephalitis associated with EBV was reported (135). Detection and removal of coexisting malignancy is important in the treatment of autoimmune encephalitis.

Interestingly, the age of patients at diagnosis differed for the various antibodies. Three cohorts were seen: age >65 years: LGI1, CASPR2, IGLON5, Ma2. Age between 50 and 60: Glycin, GAD, Yo, CV2. Age < 30 years: NMDAR, AMPAR and HE. Whether there is a pathophysiological association is not clear.

**Figure 2:**
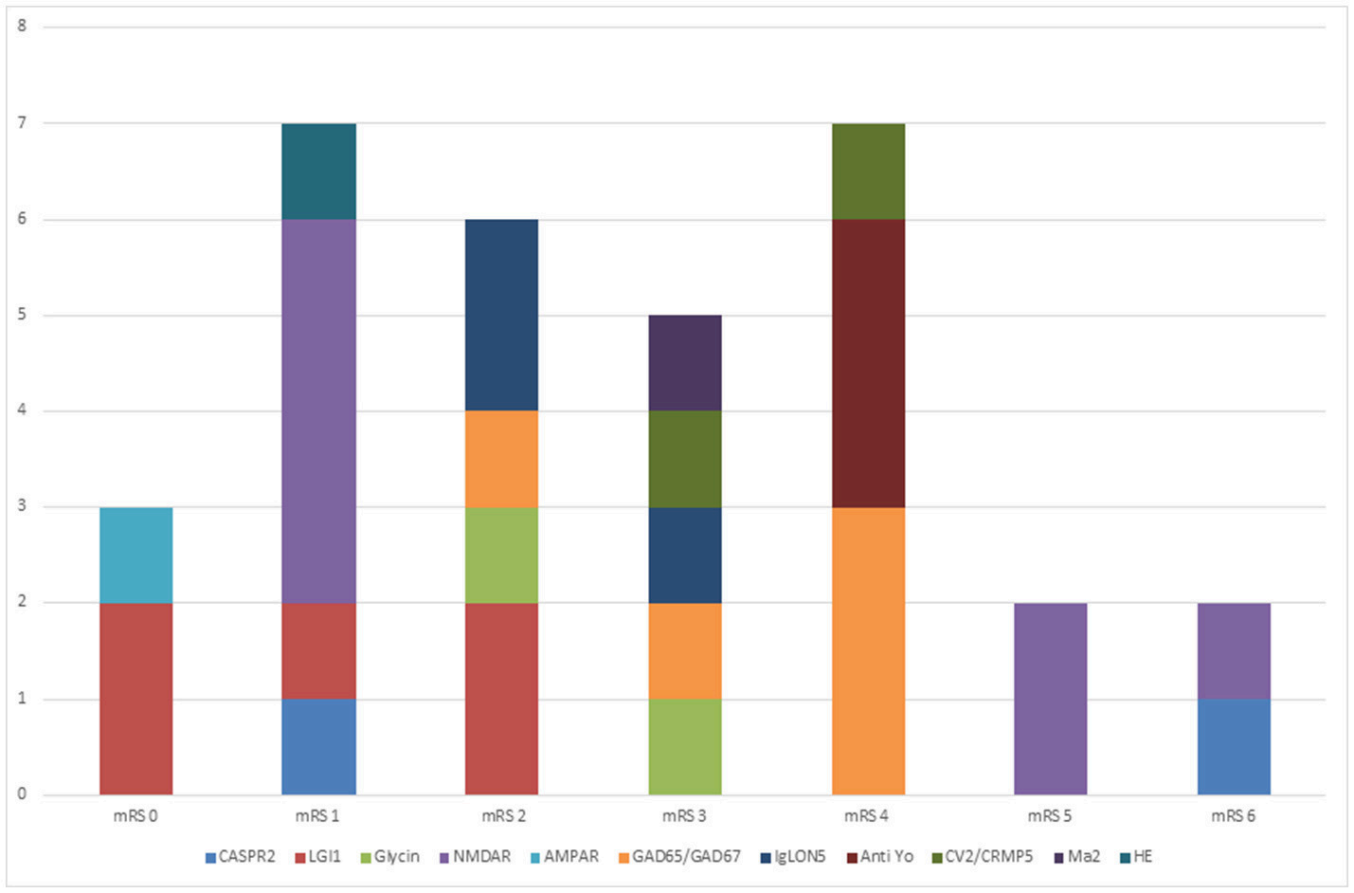
Outcome of patients with autoimmune encephalitis. This figure shows the outcome of our patients (follow up at least six months) expressed. Thirty-nine percent of our patients show no or only mild deficits (mrs ≤ 2). Twenty-seven percent show severe disability and 7% died. Best prognosis had patients with anti-LGI1 encephalitis (75% mild disability, 25% moderate disability at last follow up). All patients with onconeural antibodies have severe disability at last follow up.

Follow up data for at least 6 month is available for 29 patients. All patients with LGI1 encephalitis are independent in their daily activities and had mRS < 2, whereas for NMDAR encephalitis patients mRS ranged from 0 till 6. Sixty-seven percent of our patients with antibodies against NMDAR had a favorable outcome which is comparable to previous data that showed 81% of patients had a favorable outcome after 24 months (15). Outcome in patients with onconeuronal antibodies had a worse outcome. None of those patients were independent in daily activities. Two deaths were reported: One patient suffering from NMDAR encephalitis and one patient with CASPR2 encephalitis. See Figure 2. One of our female patients has not recovered from encephalitis despite intense immunotherapy for over 2 year now. We administered bortezomib in this case which seems promising in patients with prolonged course (136–138). Interestingly, this patient shows improvement after 22 months of treatment with walking and participation in simple conversation. This shows due to the reversible and titer-dependent internalization of the NMDAR, symptoms are reversible (48), even after that long disease duration without full recovery. If the recovery of this patient refers to bortezomib is unclear as this patient received extensive immunosuppressive therapy before.

Over the last years other treatment options have been discussed including natalizumab, azathioprine, methotrexate, mycophenolate mofetil or tocilizumab (139, 140). However, natalizumab was considered ineffective in an atypical case with NMDAR antibodies. Positive effects on seizure control, but not on cognitive deterioration was seen, when used add-on (141, 142). Treatment with natalizumab may offer a therapeutic option in autoimmune encephalitis, but as we know from multiple sclerosis treatment it should take into account years of previous immunotherapies, anti JCV antibody index but also higher risk of PML under prolonged immunosuppressive therapy (143, 144).

## Conclusion

We do show that real-life data gained in a single center is comparable with literature, although we do often stop maintenance treatment and introduce regular and close monitoring. The outcome is wide spread and depends mostly on time to diagnosis and to initiation of treatment as well as on the underlying autoantibodies and coexisting disorders, i.e., worse outcome in patients with onconeural antibodies. The frequency of autoimmune mediated encephalitis is increasing over time and more and more patients are referred from other disciplines—especially from psychiatry. This is of great importance as awareness of encephalitis mediated by autoantibodies in patients with manifold symptoms will lead to increasing numbers of testing for autoantibodies and consecutively rising numbers of patients diagnosed with autoimmune encephalitis.

We identified anti-LGi1 and anti-NMDAR encephalitis as most common causes in our cohort. Finally there are pending questions:

1. How can the identification of patients with autoimmune encephalitis in view of mostly unspecific symptoms be made easier?
2. What are the best treatment options for the various antibody associated syndromes?
3. When should treatment be escalated and when can it be terminated (as seen in one of our patients with anti-NMDAR encephalitis who show improvement to treatment after 22 months)?

Ad (1) We suggest an interdisciplinary view. Testing for antibodies should be done for sera and CSF in patients with slightest suspicion (atypical or new onset psychosis at an older age with no explanation) or a history of co-existing malignancy. Testing should be performed at institutes with proven expertise.

Ad (2) For patients with antibodies against surface ags rituximab and plasmapheresis are promising agents. For patients with onconeural antibodies tumor control is by far the best treatment option. Steroids, cyclophosphamide, or IVIGs might have some effects. Trials on immunotherapeutics for those patients should be planned. More data on best treatment options is needed. International collaborations have to be initiated. Treatment should be performed in tertiary hospitals.

Ad (3) Studies and trials have to be implemented to test for scales, biomarkers. In individual cases treatment can be stopped, still close monitoring is needed (MRI, CSF, antibody titres, neuropsychological, and clinical evaluations).

Differential diagnosis is broad and essential to be taken into account. Anamnesis, correct interpretation of the CSF, radiological assessment are the clues to appropriate diagnosis. Whereas, we are testing on slightest suspect, other clinics with not that short way to diagnostics may have to set up diagnostic pathways. Autoimmune mediated encephalitis might still be underdiagnosed, thus awareness has to be increased and testing for antibodies should be performed in sera and CSF.

## Author contributions

SM and PR are responsible for design, writing, intellectual content. All authors are responsible for intellectual content, critical review, and design.

### Conflict of interest statement

PR received speaker honoraries from Biogen, Roche, Sanofi Genzyme, Merck. PR received research support from Roche and Merck. PR served on advisory boards for Roche, Merck. The remaining authors declare that the research was conducted in the absence of any commercial or financial relationships that could be construed as a potential conflict of interest.

## Abbreviations

ADEM: acute demyelinating encephalomyelitis
CSF: cerebrospinal fluid
CBA: cell based assay
DMT1: diabetes mellitus type I
ICU: intensive care unit
MRI: magnetic resonance imaging
NFL: neurofilament light chain
NMDA-R encephalitis: N-methyl-D-aspartat-receptor encephalitis
OCB: oligoclonal bands
SPS: stiff person syndrome
SPSD: stiff person spectrum disorder.
